# Zero End-Digit Preference in Blood Pressure and Implications for Cardiovascular Disease Risk Prediction—A Study in New Zealand

**DOI:** 10.3390/jcm13226846

**Published:** 2024-11-14

**Authors:** Tanvi Chandel, Victor Miranda, Andrew Lowe, Tet Chuan Lee

**Affiliations:** 1 Institute of Biomedical Technologies, Auckland University of Technology, Auckland 1010, New Zealand; andrew.lowe@aut.ac.nz (A.L.); tet.chuan.lee@aut.ac.nz (T.C.L.); 2 Department of Mathematical Sciences, Auckland University of Technology, Auckland 1010, New Zealand; victor.miranda@aut.ac.nz

**Keywords:** blood pressure measurement errors, cardiovascular disease, risk assessment, healthcare cost

## Abstract

**Background/Objectives:** Blood pressure (BP) readings are often rounded to the nearest zero end-digit. Guidelines permit rounding to the closest 2 mmHg. This paper investigated the effect of rounding systolic blood pressure (SBP) values on the prediction of cardiovascular disease (CVD) risk among the New Zealand population. A total of 427,299 individuals received opportunistic cardiovascular disease risk assessments at primary care facilities in New Zealand. **Method:** A total of 292,122 SBP readings possessed a non-zero terminal digit. These were rounded to the nearest zero end-digit. A survival model estimating a 5-year CVD risk was applied to both datasets, i.e., with and without rounding. Hazard ratios and misclassification rates were analysed to emphasise the notable differences. Financial impact was assessed by examining healthcare expenditures. **Results:** In total, 32% of SBP values exhibited a terminal digit of zero, and 2.85% and 4.24% of men were misclassified as moderate and high risk, respectively, while approximately 3.21% of women were misclassified into the same risk categories. Likewise, 1.19% and 0.47% of men, as well as 0.62% and 0.20% of women, were misclassified into the low and moderate risk categories, respectively. **Conclusions:** Precisely measuring SBP is crucial in accurately assessing CVD risk and managing healthcare resources effectively.

## 1. Introduction

For over eight decades, the significance of precise blood pressure (BP) measurements has been widely acknowledged [1]. BP is a critical metric for health monitoring and diagnosis in healthcare settings. High BP causes around 54% of strokes and 47% of coronary heart disease worldwide [2]. BP serves as a fundamental parameter for cardiovascular disease (CVD) risk assessment [3]. It is one of the significant variables in the models used to construct CVD risk prediction equations globally.

The most accurate method for BP measurement is arterial cannulation. However, it is invasive, time-consuming, and requires skilled personnel [4]. In routine practice, BP is measured non-invasively [5]. An occluding upper arm cuff is mostly employed for intermittent non-invasive monitoring. BP readings can be assessed either manually (by auscultation of Korotkoff sounds or palpation) or mechanically (for example, using oscillometry) [5]. In clinical and research settings, a mercury sphygmomanometer is considered the traditional gold standard for non-invasive BP measurement [6]. However, these non-invasive methods introduce inaccuracies. Minor measurement errors result in the misclassification of the CVD risk of millions of individuals. This misclassification has clinical consequences [7]. The underestimation of BP by 5 mmHg would misclassify over 20 million Americans as pre-hypertensive instead of hypertensive. Conversely, overestimating BP by 5 mmHg misclassifies around 27 million people as hypertensive instead of pre-hypertensive [8]. Untreated hypertension significantly increases the risk of fatal strokes and myocardial infarctions [9]. On the other hand, overtreated hypertension can lead to adverse outcomes, such as an increased risk of hypotension, and unnecessary healthcare costs [10].

To limit the consequences of inaccurate BP measurements, the International Organization for Standardization (ISO) has established globally accepted criteria that encode acceptable limits for measurement errors and protocols to test that devices meet these criteria. ISO 81060-2:2018, for instance, outlines the standards for clinical investigations involving automated, non-invasive sphygmomanometers, a guideline that has received whole or partial approval from numerous national regulatory bodies. It outlines the procedures for testing accuracy, performance, and safety, ensuring these devices meet international standards [11]. 

Despite training in standardized procedures, BP measurements may still be subject to limitations in accuracy [12]. In a prenatal clinic based in Canada, the redefinition of a treatment threshold by a single mmHg adjustment, shifting from systolic blood pressure (SBP) > 140 mmHg to SBP ≥ 140 mmHg, resulted in a twofold increase in the percentage of patients identified as needing treatment, with the proportion escalating from 13% to 26% [13]. In a UK case–control study, the impact of terminal digit preference on disease outcomes was associated with increased mortality [14]. 

A New Zealand study examined how rounding BP measurements to zero end-digits affects patient categorization for pharmacological management in primary care. It found that about 64.4% of SBP values ended in zero [15]. Measurements rounded to the nearest even number, as recommended, should have around 20% zero end-digits [16]. The study employed three distinct risk prediction algorithms sourced from different countries, each incorporating a range of risk factors. One of these algorithms is based on the US population, which was developed from data obtained from the Framingham Heart Study [17]. The remaining two algorithms were based on the 2004 British Hypertension Society (BHS) guidelines (BHS-IV) [18] and the 2005 Joint British Societies’ (JBS) guidelines (JBS2) [19]. The primary objective of this NZ-based study [15] was to assess the consequences of rounding BP measurements to zero end-digits when applying diverse risk prediction models from different countries within primary care in the context of NZ. The results showed the misclassification of 1 in 41 patients, potentially altering treatment decisions. Under the JBS2 guidelines, 1 in 19 would be misclassified, and under the BHS-IV guidelines, 1 in 12, primarily leading to increased treatment. At the time of this study, NZ did not have a locally developed CVD risk prediction equation.

Initially, NZ was using the Framingham Risk Score for their CVD risk management guidelines. However, these equations were developed targeting the US population, which might result in an incorrect estimation of risk for other populations with diverse ethnic backgrounds. This led to the development of a region-specific CVD risk equation [20]. In 2018, NZ introduced its CVD risk prediction equation known as the PREDICT-1 equation [21]. It estimates an individual’s 5-year CVD risk and is specifically tailored to the NZ population from a study cohort also known as PREDICT. The five-year CVD risk was used to guide decision making for primary prevention. The New Zealand CVD risk management guidelines recommend the selection of a 5-year risk assessment [22] instead of the more commonly used 10-year risk period, as most trials of CVD risk reduction have about 5 years of follow-up [23,24]. Table 1 summarises the recommended interventions, goals, and follow-up based on CVD risk assessment for clinicians relative to the three risk categories. These risk categories are used in current CVD management guidelines in NZ: <5% (low-risk group), 5–15% (moderate-risk group), and >15% (high-risk group). 

Patients are categorised into three risk groups based on the data collected from the PREDICT study cohort. However, the potential impact of rounding BP measurements on decisions related to pharmacological treatment, as guided by the PREDICT-1 equation, has not been fully explored. This paper investigates the misclassification arising from the rounding practices of SBP and evaluates its effect on determining eligibility for CVD risk management through medication. 

Our primary focus is on the potential consequences of misclassifying moderate-risk patients as low-risk. Misclassification in this context leads to significant undertreatment, as low-risk patients typically receive recommendations for lifestyle modifications rather than pharmacological interventions. While high-risk patients receive comprehensive medical treatment, many moderate-risk patients are also prescribed medications based on their clinical evaluation. However, those misclassified in the low-risk category are at greater risk of not receiving the necessary medical intervention. Additionally, this paper also examines how these misclassifications due to rounding impact the overall healthcare costs within the NZ healthcare system. 

## 2. Methods

### 2.1. Study Population

PREDICT is an ongoing, open cohort study for the NZ population. Cardiovascular risk is composed of various events, including ischemic or haemorrhagic cerebrovascular events, peripheral vascular disease, and congestive heart failure. PREDICT is embedded into practice management software systems facilitating the healthcare needs of at least a third of the country’s residents [21]. The cohort under examination is gender specific. The study period spans from the initial assessment until the earliest of the following events: hospital admission, the conclusion of the follow-up period, and death due to CVD or other causes. 

In this paper, risk profiles were gathered for every person whose first risk assessment was conducted between October 2004 and December 2018 within routine healthcare settings. The 5-year CVD risk score, estimated as the percentage risk of an individual having a CVD event in 5 years, is evaluated based on the risk factors outlined in Table 2. Specifically, for each patient, the 5-year absolute risk of a CVD event was obtained by applying ‘PREDICT-1’ [21], given by

(1) $$Risk\;\left(\%\right)=\left(1-{S_{0}}^{e^{\left(\sum \beta_{1}z_{1}+\beta_{2}z_{2}+\ldots \ldots +\beta_{p}z_{p}\right)}}\right)*100,$$

where $S_{0}$ is the baseline survival function, $\beta_{i}$ coefficients are the beta estimates obtained for each variable mentioned in Table 1, and $z_{i}$ are the corresponding risk factors.

The coefficients are estimated by applying the Cox Proportional Hazard Model (Cox PH). The Cox PH model is [26] expressed as

(2) $$h\left(t\right)={\;h}_{0}\left(t\right)*\exp\left(\beta_{1}z_{1}+\beta_{2}z_{2}+\ldots \ldots +\beta_{p}z_{p}\right),$$

where

*h*(t) is determined by p covariates ($z_{1},z_{2},\ldots .,\;z_{p}$). The impact of each covariate is measured by their respective coefficients ($\beta_{1},\beta_{2},\ldots ..,\beta_{p}$).

$h_{0}\left(t\right)$ is the baseline hazard when all the covariates are zero at time t, and ‘t’ in *h*(t) indicates that the hazard will change over time. 

### 2.2. Study Design

For patients whose SBP did not end in zero, a second SBP value was assigned by rounding the original measurement to the nearest zero end-digit. End-digits one to four were rounded down, and six to nine were rounded up. The end-digits of five were randomly rounded up or down with equal probability. This method replicates common rounding practices observed in routine healthcare for manually recorded measurements [15]. 

**Figure 1:**
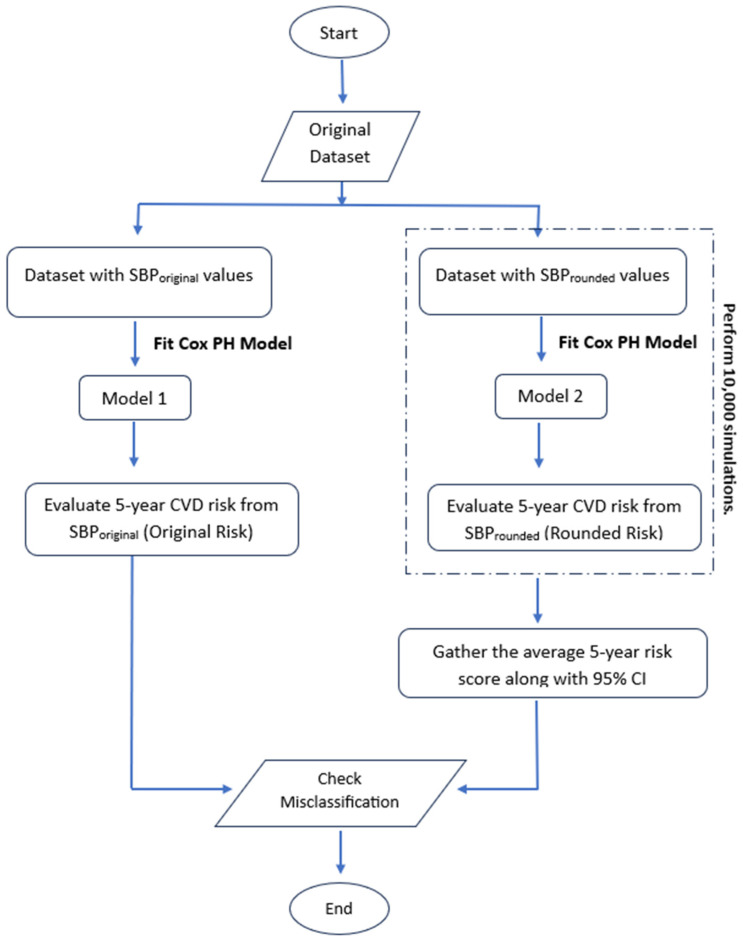
Process to evaluate the misclassification rate by introducing a new model with rounded SBP values.

The impact of rounding SBP values is assessed by examining how rounding to the nearest zero end-digit affects CVD risk prediction and alters the model. Figure 1 illustrates the overall design to study the misclassification rates when a new survival model is fitted on the existing dataset with rounded SBP values. The detailed explanation of the design and simulation study conducted is as follows:

- Simulations were conducted for men and women separately. A CoxPH model (Model 1) was fitted using a subset of patients whose original BP (SBP_original_) was measured without a zero end-digit. The beta coefficients derived from Model 1 were used to calculate the 5-year CVD risk (Original Risk) for each individual, categorizing patients into three distinct CVD risk categories.
- This subset of non-zero SBP values were rounded to the nearest zero end-digit (SBP_rounded_). A new model (Model 2) was then fitted on this dataset. The coefficients of Model 2 were used to estimate the 5-year CVD risk (Rounded Risk). This process was repeated 10,000 times to account for variability and ensure robustness in the findings. For each simulation, individuals were categorized into one of three CVD risk categories based on their calculated 5-year risk. The total number of individuals in each risk category was recorded. The average number of individuals in each risk category across all 10,000 simulations was then computed, along with the 95% confidence intervals (CIs) to quantify the uncertainty around the risk classifications.
- The model estimates in terms of hazard ratio (HR) were compared for both the models, i.e., Model 1 and Model 2, by conducting a paired T-test to check significant differences. The relative difference between the two models was also studied, which was evaluated using Equation (3). The classification results obtained from SBP_original_ and SBP_rounded_ were compared. This comparison allowed for an assessment of the extent of misclassification and the potential impact of rounding SBP values on the accuracy of CVD risk prediction.

(3) $$Relative\;Difference\;=\frac{{\;\mathrm{HR}}_{Model\;2}-{\;\mathrm{HR}}_{Model\;1}}{{\mathrm{HR}}_{Model\;1}}*100$$

To account for device calibration to the nearest 2 mmHg, we estimate the proportion of rounding in the whole cohort by dividing the data into three groups: (1) non-zero end-digit SBP values, (2) true zero-end digits (20% prevalence), and (3) zero end-digits likely due to rounding. Using this estimate, we apply stratified sampling to select the same percentage of patients from the non-zero group and round their SBP values. Stratified sampling ensures proportional representation across quantiles based on key CVD factors minimizing bias in risk profiles. This method accounts for variations across different risk profiles, thereby reducing the risk of over- or underrepresentation. After adjusting the data through rounding, we refit the Cox PH model using the updated dataset. The same simulation approach, as previously outlined in this paper, is then applied to assess the impact of rounding on CVD risk predictions and model performance. 

To study the financial impact, a comprehensive cost assessment is conducted for key resources used for CVD risk assessment. The cost associated with these resources were gathered from multiple official sources that provide the latest cost schedules and medication prices relevant to CVD risk assessment and are presented below:

1. Data Sources:
  - Government Databases—Cost Resource Manual and Community Pharmaceutical Schedule provided by PHARMAC [27,28].
  - Ministry of Health—Cardiovascular Disease Risk Assessment and Management for Primary Care document highlighting key variables for CVD risk assessment [25].
2. Cost Variables:
  - Consultation Fees
  - Diagnostic Procedures
  - Medications

Based on these resources, the average cost in 5 years associated with CVD risk management has been estimated. Additionally, a minimum and maximum cost range was estimated to account for variations.

## 3. Results

Our dataset includes BP measurements from 427,299 individuals in the PREDICT cohort. Among these, men (n = 241,036) reported an average age of 51.29 years (SD = 10.07 years), while women (n = 186,263) had an average age of 55.85 years (SD = 8.87 years). For men, 32.16% of the SBP values ended in a zero, while for women, this figure was 30.96%. Overall, approximately 32% of the total dataset consisted of SBP values ending in zero, with the remaining having non-zero end-digits.

**Figure 2:**
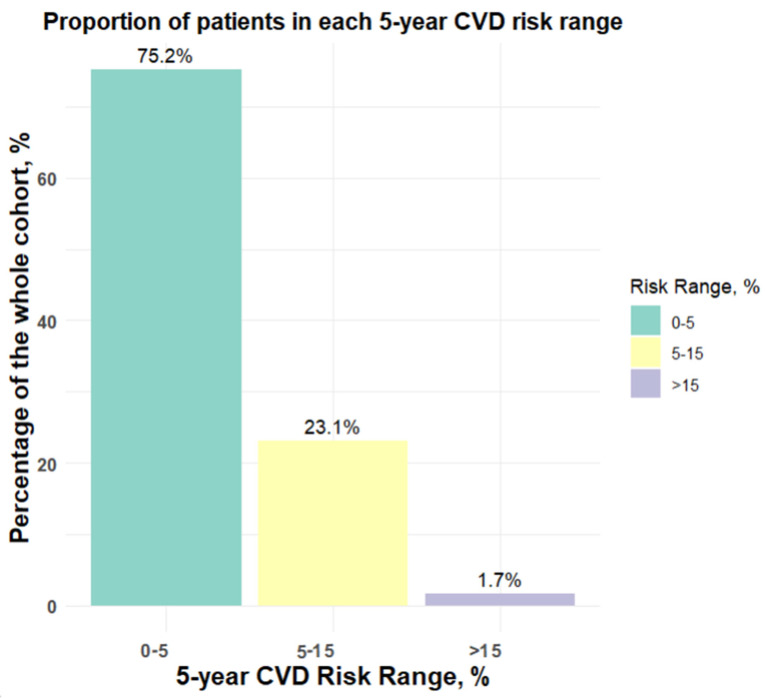
Proportion of patients in each 5-year CVD risk range.

Figure 2 shows the distribution of people across three CVD risk categories. The largest group of people falls into the lowest risk category, that is, having less than 5% risk of developing CVD within the next five years. A smaller proportion of people fall into the 5–15% risk group, with the smallest proportion in the high-risk category, which is >15%. 

Table 3 gives the demographics and risk factors pertaining to the SBP end-digit groups for men and women. After excluding individuals with a history of CVD or those with SBP values ending in zero, the study resulted in a final cohort of 163,528 men and 128,594 women. 

**Figure 3:**
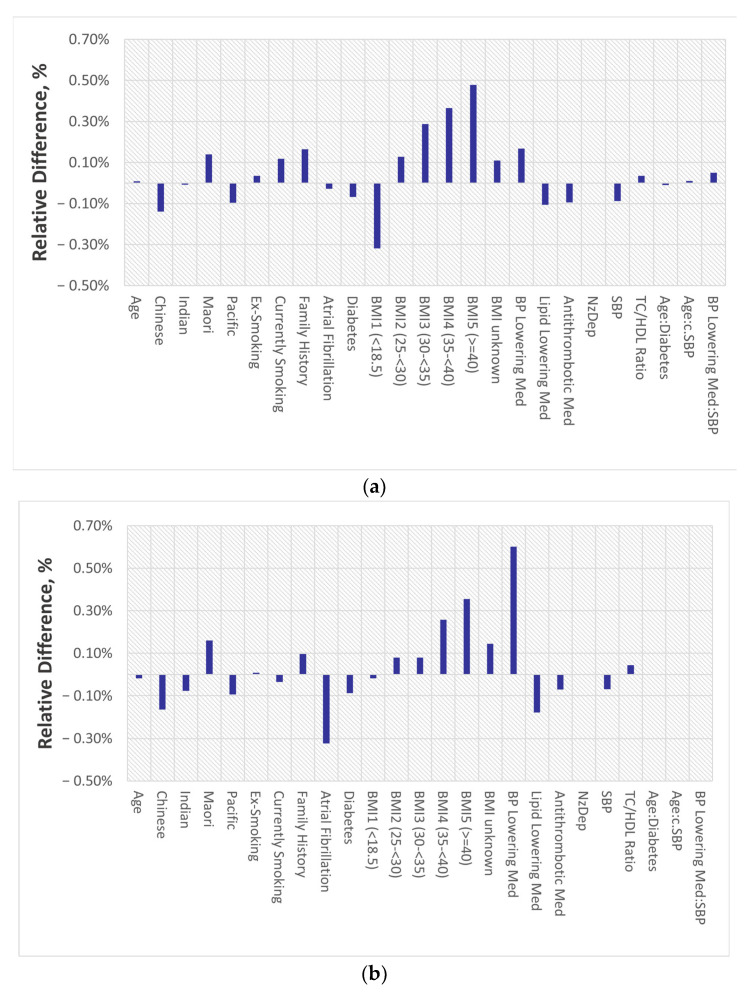
Relative change in the hazard ratio due to rounding for (**a**) men and (**b**) women, respectively.

While comparing the estimates for Model 1 and Model 2 for both men and women, no significant differences were observed in the HRs with a *p*-value above 0.05. The highest relative difference for men and women was about 0.60%. This relative difference is illustrated in Figure 3. Further details regarding the hazard ratios of the models are presented in Table A1 and Table A2 (Appendix A). 

**Figure 4:**
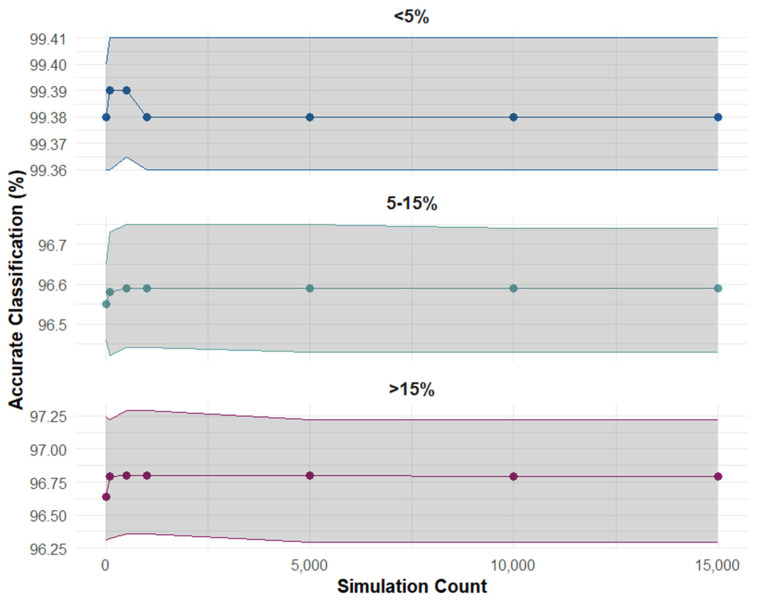
Accurate classification across risk groups by simulation count for women.

To evaluate the stability and reliability of the Cox model in categorising individuals into distinct risk categories, we performed a series of simulations with varying sample sizes (ranging from 10 to 15,000). The analysis for women presented in Figure 4 indicated that within roughly 5000 iterations, the variability in classification accuracy stabilized, as demonstrated by minimal fluctuations in the percentage of correct classifications and overlapping 95% confidence intervals. The same pattern was observed for men as well. The results suggest that beyond 5000 simulations, additional runs contribute little to overall accuracy improvement. We increased the simulation count to 10,000 for the analysis, to enhance the robustness of our estimates, mitigate potential random fluctuations associated with smaller counts, and strengthen the robustness of our conclusions.

**Figure 5:**
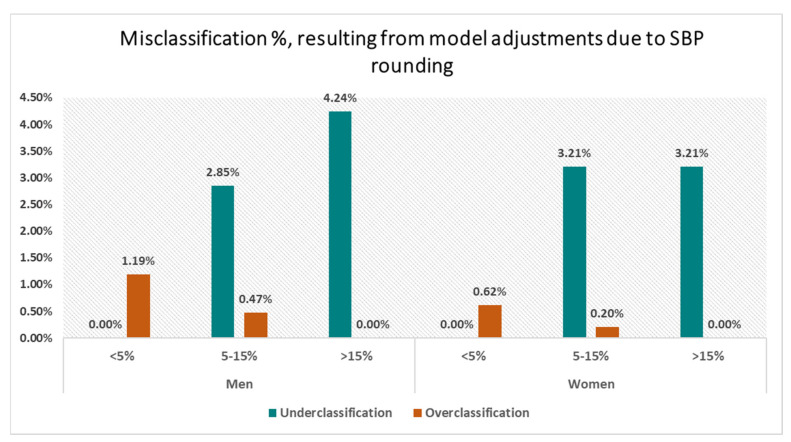
Misclassification plots due to rounding per risk group for women and men.

Figure 5 shows the plots for misclassification in the three risk groups due to rounding SBP for men and women. More under classification is noted than overclassification, with a higher proportion of misclassification noted for men compared to women. The plots show that approximately 4.24% of high-risk men and 3.21% of high-risk women are misclassified into lower risk categories. In total, 1.19% of men are overclassified into the moderate-risk group, and 0.47% into the high-risk group. For women, 0.62% are overclassified into the moderate-risk group, and 0.20% into the high-risk group. Table A3 in Appendix B provides the 95% CI values for the misclassification rates for each risk group.

While there is minimal deviation in the HRs for all the predictors used in Equation (1), there is still a notable misclassification in cardiovascular risk groups when rounding the SBP values to the nearest zero end-digit. The average number of people misclassified into different risk groups are mentioned in Table 4. For instance, among women in our sample, 670 moderate-risk patients were misclassified as low-risk, leading to undertreatment, whilst 687 low-risk patents were misclassified as moderate-risk, which could lead to overtreatment. Similarly, we found that 1291 men previously in the moderate-risk group were misclassified into the low-risk group, and 1354 men initially in the low-risk category were incorrectly classified as moderate-risk. 

The results in Table 4 are generated by rounding all non-zero end-digit SBP values. As discussed earlier, around 32% of the data have zero end-digits. Assuming device calibration to the nearest 2 mmHg, around 12% of data were rounded to the nearest zero end-digit. After adjusting for the entire cohort, on average, 304 patients (193 men and 111 women) were undertreated, while 308 patients (201 men and 107 women) were overtreated due to misclassification. 

The results from stratified sampling across 100 iterations showed minimal deviation in the misclassification rates, with a maximum variation of only 0.001 and overlapping confidence intervals. The average estimates of the misclassification rate are presented in Table A4 in Appendix B. No significant changes were observed in the overall survival model when generalized to the whole cohort. Further details on the hazard ratios from the model can be found in Table A1 and Table A2 in Appendix A.

## 4. Financial Impact

The cost data were sourced from the PHARMAC Cost Resource Manual [27]. This manual provides key pricing information relevant to the funding of pharmaceuticals, including consultation and diagnostic costs. The gathered data, along with minimum and maximum cost ranges, are presented in Table 5. Table 5 outlines the total consultation and diagnostic costs (in NZD) over a 5-year period for the three cardiovascular risk groups. The estimates account for the differences in follow-up assessments specific to each risk group, as mentioned in Table 1. They also reflect the variations in the frequencies of healthcare interventions required for effective risk management across different risk levels.

Additionally, medication cost estimates were derived from various reports on the type of medications recommended in New Zealand. These estimates were based on the community pharmaceutical schedule [28], which lists all medications funded for New Zealand residents. Table 6 details the daily per-dose cost of three commonly prescribed medications for managing CVD: statins, antihypertensives, and aspirin [29,30,31]. 

The medications are typically prescribed for long-term CVD prevention [32,33,34]. For individuals with a CVD risk of less than 5%, lifestyle changes are primarily recommended, and medication is not generally prescribed. In contrast, for those with a risk greater than 15%, the total estimated cost in 5 years per person ranges from NZD 1198 to NZD 5908, reflecting variations in the type and dosage of medications prescribed. 

For moderate-risk patients, there is evidence that statins and antihypertensives are recommended, as they have been shown to provide significant benefits in reducing cardiovascular events [35,36]. The addition of antithrombotics is considered beneficial only for secondary prevention in those with a higher CVD risk (≥15%), where the benefits generally outweigh the risks despite the potential for side effects like gastrointestinal bleeding and ulceration [32]. However, the decision to prescribe additional medications ultimately depends on the physician’s assessment of the patient’s overall health profile, including comorbidities and specific risk factors [25]. For cost estimation of the moderate-risk group, the prices of statins and antihypertensives are used, as they are commonly prescribed medications. The average estimated total cost in 5 years is NZD 1255.38 per person, ranging from NZD 766 to NZD 4669. This approach conservatively estimates cost, as the actual cost could vary based on medication type and the number of medications prescribed.

For around 2008 overclassifications into a moderate-risk category for men and women, as shown in Table 4, expenses of approximately NZD 1.57 million will be incurred, with expenses ranging up to NZD 8.2 million. Additionally, the misclassification of 1961 men and women into the low-risk category leads to undertreatment, which significantly impacts hypertension management and increases the likelihood of adverse outcomes, such as cardiovascular events, renal dysfunction, and all-cause mortality [37]. Undertreated patients are at a higher risk of experiencing these adverse events, contributing to increased healthcare costs. The inpatient cost for managing cardiovascular events ranges from NZD 1200 for a hospital medical ward to NZD 5500 per patient for an ICU, expenses that could largely be avoided through accurate risk assessment and timely intervention [27]. 

## 5. Discussion

The practice of recording BP with a zero end-digit, known as “zero end-digit preference”, is common in primary care, particularly during routine measurements [38]. This poses significant implications when healthcare professionals employ equations like the PREDICT-1 equation to estimate a patient’s 5-year CVD risk [39]. It was assumed that the measurement devices being used were standard mercury auscultatory sphygmomanometers as they remain the gold standard for non-invasive BP measurement due to their accuracy compared to other devices. The mercury sphygmomanometer continues to serve as the reference standard for validating other devices in development and clinical studies as per almost all the international guidelines [11,40]. Thus, it becomes crucial to understand the impact of rounding, a prevalent practice in clinical settings. As per survey data in 2007, it was identified that using mercury sphygmomanometers is almost universal in these clinical practices in NZ [15]. However, there has been no information gathered since then regarding the type of measurement device used, emphasizing the need for improved data collection in the future. 

This work used a substantial patient cohort sourced from over 400 general practitioners (GPs) and analysed data from standard primary care settings, where healthcare providers measured SBP during routine visits. It was assumed that individuals with non-zero SBP values had their BP recorded more accurately, given the lack of evidence for rounding to other end-digits [15]. The analysis estimates the upper-bound impact of rounding errors by rounding non-zero SBP values and also includes a generalized version for the entire cohort.

The prevalence and implications of zero end-digit preference have been addressed in previous studies. Research involving 85,000 BP measurements in patients with ischaemic heart disease in England discovered that zero end-digit preference accounted for 64% of SBP and 59% of DBP measurements [41]. A study demonstrated that the error variance of the associations between BP and both BMI and age was higher when zero end-digit preference for BP readings was employed in comparison to unbiased readings [39]. 

The observed effect of BP rounding on CVD risk misclassification highlights the importance of establishing measures to minimise rounding inaccuracies in clinical assessments. Standardising BP measurement techniques is an essential initial step, prioritising the training of clinical personnel to accurately document readings. Many large surveys including NHANES have advocated appropriate certification and periodic re-certification as a means to mitigate these errors [42]. Transitioning to digital sphygmomanometers, where feasible, can reduce human error and provide consistency across providers. The use of automated devices has demonstrated efficacy in reducing zero end-digit preference in studies like the Hypertension Optimal Treatment (HOT) and Anglo-Scandinavian Cardiac Outcomes Trial (ASCOT) [39]. It is recommended to revise clinical guidelines to consider the effects of BP rounding on CVD risk assessment, in addition to conducting regular educational sessions that highlight the importance of precise measurement in risk classification.

The evidence shows a higher proportion of misclassifications in lower risk categories for both men and women, a trend that may be influenced by the greater representation of individuals within the low-risk group among NZ’s population. This study demonstrated that BP measurements were more frequently rounded down than up at various stages of hypertension. The propensity to round down may result in a higher frequency of individuals being underclassified instead of overclassified into lower risk categories, as BP demonstrates a positive correlation with CVD risk [43]. This influence can potentially lead to the mistreatment of an individual when considering other relevant factors [44]. If undertreated, the individual might not be prescribed necessary medications or lifestyle changes to manage their actual higher risk, leading to a delay in necessary treatment, which might increase the likelihood of adverse CVD events. Conversely, overtreatment may subject individuals to unnecessary interventions and frequent monitoring, leading to inefficiencies in healthcare resource allocation [45,46]. While estimating cost, it was assumed that all individuals in the moderate-risk group were only prescribed statins and antihypertensives. Despite being a conservative estimate, it amounted to a large financial burden. With additional information, the estimate might be much higher. Hence, accurate classification is foremost in CVD risk management. 

Research highlights the importance of achieving treatment targets to prevent cardiovascular events. Maintaining an optimal BP control, as per the 2017 ACC/AHA guidelines, could prevent 71.9 cardiovascular events per 1000 treated individuals over 10 years [47]. A failure to initiate appropriate interventions or adhere to recommended treatments increases the risk of cardiovascular events by 20% and mortality by 35%. This leads to extended hospital stays and a heavier long-term healthcare burden. Accurate risk classification and early management are therefore essential in reducing both clinical and financial strain [48].

The likelihood of BP rounding may be dependent on certain confounding factors, such as socioeconomic status, e.g., high NZDep scores, or access to better healthcare facilities. However, due to the lack of data or research on these specific factors, it was not feasible to account for them in this study. This limitation emphasises the need to conduct future research to investigate the potential impact of these variables on BP rounding practices, thereby enabling a more precise generalization and adjustment for potential confounders.

We acknowledge that there are various other sources of BP measurement errors, such as device calibration issues, repeated measurements and patient factors like white coat hypertension or improper cuff size, which can all influence treatment decisions [10,49]. However, this study specifically focuses on the impact of rounding errors on treatment thresholds established by the Ministry of Health in NZ [25]. The results provide additional insights into the existing body of knowledge, highlighting that while they are not the sole determinant, they are an important factor in clinical decision making. Future research should also consider the additional sources of error to provide a more comprehensive understanding of the challenges in accurate BP measurement and improve clinical outcomes.

The interpretation of our study findings is particular to the New Zealand context, as we are investigating the impact of rounding BP on CVD risk prediction using New Zealand-based guidelines and data. These outcomes may differ in various nations due to variations in attributes, such as ethnicity, socioeconomic level, and healthcare practices. Furthermore, our healthcare cost estimate is based on cost schedules from PHARMAC, a New Zealand government agency, which may vary from cost structures and insurance procedures in other nations. Future research in other regions may yield comparative insights and evaluate the relevance of these findings across varied healthcare systems.

## 6. Conclusions

This research evaluated the impact of rounding by applying it to the entire subset of non-zero SBP records and then generalizing the results to the entire cohort. Although the overall model showed only slight variations in both cases, notable misclassification was still observed. This misclassification contributes to an increased healthcare burden, as it affects the accuracy of risk classification, potentially leading to inappropriate medical interventions. As a result, patients may not receive the necessary treatment, ultimately compromising their health outcomes and increasing the likelihood of adverse cardiovascular events. Potential solutions to reduce rounding errors emphasise the need for education programmes highlighting the impact of rounding for healthcare practitioners with periodic retraining in BP measurement. Implementing these strategies can improve the reliability of CVD risk assessment tools, ultimately supporting better clinical outcomes and more informed decision making for both healthcare providers and policymakers.

## Figures and Tables

**Table 1:** Recommended interventions, goals, and follow-up based on CVD risk assessment [25].

Risk Category	5-Year CVD Risk	Recommended Intervention and Goals	Follow-Up
Low Risk	<5%	▪ Medication management is typically not advised. ▪ Emphasis is placed on lifestyle changes, such as improving diet, increasing exercise, and quitting smoking.	Reassessment in 5–10 years
Moderate Risk	5–15%	▪ The use of lipid-lowering and blood-pressure-lowering medications is advised for managing CVD risk in patients. ▪ Particular attention is given to prescribing medications for individuals at the higher end of this risk range.	Reassessment every 2–5 years
High Risk	>15%	▪ Medication therapy with lipid-lowering and blood-pressure-lowering agents is highly advised. ▪ This group is regarded as having a risk level similar to individuals with established CVD.	Annual reassessment

**Table 2:** Risk factors obtained from the PREDICT study.

Risk Factors	Type
Age (centred)	Numeric
Ethnicity	European
	Maori
	Pacific
	Indian
	Chinese or other Asian
New Zealand Deprivation Index (NzDep)	1 (least deprived)
	2
	3
	4
	5 (most deprived)
Ex-smoker	0 = No 1 = Yes
Current smoker	0 = No 1 = Yes
Family history of premature CVD	0 = No 1 = Yes
Atrial fibrillation	0 = No 1 = Yes
Diabetes	0 = No 1 = Yes
Systolic blood pressure (SBP, centred)	0 = No 1 = Yes
Total cholesterol to high-density lipoprotein cholesterol (TC:HDL) ratio (centred)	0 = No 1 = Yes
BMI	normal
	underweight
	overweight
	obesity class 1
	obesity class 2
	obesity class 3
	bmi unknown
On BP-lowering medication	0 = No 1 = Yes
On lipid-lowering medication	0 = No 1 = Yes
On either antiplatelet or anticoagulant medications	0 = No 1 = Yes

**Table 3:** Overview of the demographics and risk factors of the SBP end-digit groups among men and women.

Risk Factors	MEN			WOMEN		
	SBP End-Digit (%)			SBP End-Digit (%)		
	Zero	Other	Total	Zero	Other	Total
**Self-Identified Ethnicity**						
European	29.30%	70.70%	138,195	28.50%	71.50%	103,922
Maori	32.30%	67.70%	31,473	30.30%	69.70%	27,141
Pacific	37.30%	62.70%	35,083	35.20%	64.80%	27,660
Indian	35.70%	64.30%	21,500	34.90%	65.10%	14,100
Chinese or other Asian	41.60%	58.40%	14,785	38.40%	61.60%	13,440
**NZDep quintile**						
1 (least deprived)	31.00%	69.00%	53,260	30.60%	69.40%	41,210
2	31.30%	68.70%	47,818	30.00%	70.00%	36,545
3	31.30%	68.70%	42,676	30.20%	69.80%	33,131
4	32.70%	67.30%	44,152	31.70%	68.30%	34,078
5 (most deprived)	34.40%	65.60%	53,130	32.20%	67.80%	41,299
**Ex-smoker**						
Yes	29.60%	70.40%	44,540	27.70%	72.30%	28,929
No	32.70%	67.30%	196,496	31.60%	68.40%	157,334
**Current smoker**						
Yes	33.70%	66.30%	40,287	30.90%	69.10%	23,798
No	31.90%	68.10%	200,749	31.00%	69.00%	162,465
**Family history of premature cardiovascular disease**						
Yes	28.10%	71.90%	23,452	27.40%	72.60%	21,969
No	32.60%	67.40%	217,584	31.40%	68.60%	164,294
**Atrial fibrillation**						
Yes	28.00%	72.00%	4060	26.70%	73.30%	1982
No	32.20%	67.80%	236,976	31.00%	69.00%	184,281
**Diabetes**						
Yes	31.80%	68.20%	24,063	32.00%	68.00%	22,475
No	32.20%	67.80%	216,973	30.80%	69.20%	163,788
**Blood**-**pressure-lowering medication**						
Yes	28.20%	71.80%	49,261	28.40%	71.60%	50,728
No	33.20%	66.80%	191,775	31.90%	68.10%	135,535
**Lipid-lowering medication**						
Yes	30.10%	69.90%	40,013	30.40%	69.60%	31,478
No	32.60%	67.40%	201,023	31.10%	68.90%	154,785
**Antithrombotic medication**						
Yes	29.80%	70.20%	24,384	30.20%	69.80%	19,186
No	32.40%	67.60%	216,652	31.10%	68.90%	167,077
**BMI**						
normal	33.20%	66.80%	41,862	31.40%	68.60%	44,654
underweight	35.90%	64.10%	781	31.50%	68.50%	1869
overweight	30.80%	69.20%	82,268	30.00%	70.00%	46,082
obesity class 1	30.90%	69.10%	46,854	30.20%	69.80%	29,691
obesity class 2	30.90%	69.10%	17,787	30.10%	69.90%	16,621
obesity class 3	32.20%	67.80%	10,070	30.40%	69.60%	13,918
bmi unknown	35.60%	64.40%	41,414	33.10%	66.90%	33,428

**Table 4:** Misclassification count for men and women along with 95% CIs.

		Rounded Risk			
	Original Risk	<5%	5–15%	>15%	Total
Women	<5%	105,629 (105,599–105,656)	654 (627–684)	0	106,283
	5–15%	670 (639–703)	20,196 (20,162–20,227)	43 (35–51)	20,909
	>15%	0	45 (39–52)	1357 (1350–1363)	1402
Men	<5%	112,257 (112,214–112,296)	1354 (1315–1397)	0	113,611
	5–15%	1291 (1250–1334)	43,839 (43,784–43,888)	215 (193–241)	45,345
	>15%	0	194 (181–208)	4378 (4364–4391)	4572

**Table 5:** Estimated cost breakdown for consultation and diagnostic resources used in CVD risk assessment.

	Frequency of Assessment (in 5 Years)	Minimum Cost	Cost	Maximum Cost
GP Visit		NZD 80.00	NZD 80.00	NZD 80.00
Nurse Visit		NZD 40.00	NZD 40.00	NZD 40.00
Lipid Profile Test		NZD 11.64	NZD 22.89	NZD 46.50
Blood Glucose Test		NZD 15.12	NZD 24.27	NZD 39.50
Electrocardiogram (ECG)		NZD 60.00	NZD 68.80	NZD 85.00
Cost (Annual)		NZD 206.76	NZD 235.96	NZD 291.00
Cost (in 5 years)				
Risk Category: <5%	2	NZD 413.52	NZD 471.92	NZD 582.00
Risk Category: 5–15%	3	NZD 620.28	NZD 707.88	NZD 873.00
Risk Category: >15%	5	NZD 1033.80	NZD 1179.80	NZD 1455.00

**Table 6:** Estimated cost breakdown for medications prescribed for CVD management.

	Minimum Cost	Cost	Maximum Cost
Statins	NZD 0.05	NZD 0.05	NZD 0.05
Antihypertensives	NZD 0.03	NZD 0.25	NZD 2.03
Antithrombotics	NZD 0.01	NZD 0.14	NZD 0.36
Cost (Annual)	NZD 32.85	NZD 160.60	NZD 890.60
Cost (in 5 years)			
Risk Category: <5%	NA	NA	NA
Risk Category: 5–15%	NZD 146.00	NZD 547.50	NZD 3796.00
Risk Category: >15%	NZD 164.25	NZD 803.00	NZD 4453.00

## Data Availability

The data presented in this study were made available by the VAREANZ Data Sovereignty Group, in agreement with the New Zealand Ministry of Health. Data availability requests should be directed to rt.jackson@auckland.ac.nz.

## Appendix A

**Table A1:** Hazard ratios for the original model and the rounded model for men.

Variables	Original Dataset	Full Rounding	Generalised (12% Rounding)
Age	1.0715	1.0716	1.0715
Chinese	0.6508	0.6499	0.6509
Indian	1.1849	1.1848	1.1848
Maori	1.3663	1.3682	1.3658
Pacific	1.2497	1.2485	1.2495
Ex-Smoking	1.1167	1.1171	1.1167
Currently Smoking	1.7809	1.783	1.7811
Family History	1.1598	1.1617	1.1606
Atrial Fibrillation	1.7895	1.789	1.7915
Diabetes	1.6338	1.6327	1.6344
BMI1 (<18.5)	1.756	1.7504	1.7563
BMI2 (25–<30)	0.9345	0.9357	0.9345
BMI3 (30–<35)	0.9754	0.9782	0.9753
BMI4 (35–<40)	1.0955	1.0995	1.0957
BMI5 (≥40)	1.4224	1.4292	1.4226
BMI unknown	0.9053	0.9063	0.9054
BP Lowering Med	1.309	1.3112	1.3081
Lipid Lowering Med	0.9425	0.9415	0.9426
Antithrombotic Med	1.0589	1.0579	1.0588
NzDep	1.0597	1.0597	1.0597
SBP	1.0188	1.0179	1.0187
TC/HDL Ratio	1.1422	1.1426	1.1423
Age: Diabetes	0.9845	0.9844	0.9844
Age: c.SBP	0.9994	0.9995	0.9994
BP Lowering Med: SBP	0.9963	0.9968	0.9964

**Table A2:** Hazard ratios for the original model and the rounded model for women.

Variables	Original Dataset	Full Rounding	Generalised (12% Rounding)
Age	1.0761	1.0759	1.0761
Chinese	0.6712	0.6701	0.6707
Indian	1.166	1.1651	1.1655
Maori	1.6181	1.6207	1.6185
Pacific	1.2874	1.2862	1.2875
Ex-Smoking	1.1409	1.141	1.1412
Currently Smoking	1.9554	1.9547	1.956
Family History	1.0323	1.0333	1.0324
Atrial Fibrillation	2.5997	2.5913	2.5995
Diabetes	1.5993	1.5979	1.5993
BMI1 (<18.5)	1.6933	1.693	1.6945
BMI2 (25–<30)	0.9985	0.9993	0.9992
BMI3 (30–<35)	0.9977	0.9985	0.9983
BMI4 (35–<40)	1.0474	1.0501	1.0487
BMI5 (≥40)	1.377	1.3819	1.3789
BMI unknown	1.0377	1.0392	1.038
BP Lowering Med	1.414	1.4225	1.415
Lipid Lowering Med	0.9578	0.9561	0.9575
Antithrombotic Med	1.1394	1.1386	1.1388
NzDep	1.1035	1.1035	1.1036
SBP	1.0189	1.0182	1.0187
TC/HDL Ratio	1.1317	1.1322	1.1318
Age: Diabetes	0.9856	0.9856	0.9856
Age: c.SBP	0.9995	0.9995	0.9995
BP Lowering Med: SBP	0.9925	0.9925	0.9926

## Appendix B

**Table A3:** Misclassification rate for men and women along with the 95% CI (Full Rounding).

		Rounded Risk		
	Original Risk	<5%	5–15%	>15%
Women	<5%	99.38% (99.36–99.41%)	0.62% (0.59–0.64%)	0.00%
	5–15%	3.21% (3.06–3.36%)	96.59% (96.43–96.74%)	0.20% (0.17–0.24%)
	>15%	0.00%	3.21% (2.78–3.71%)	96.79% (96.29–97.22%)
Men	<5%	98.81% (98.77–98.84%)	1.19% (1.16–1.23%)	0.00%
	5–15%	2.85% (2.76–2.94%)	96.68% (96.56–96.79%)	0.47% (0.43–0.53%)
	>15%	0.00%	4.24% (3.96–4.55%)	95.76% (95.45–96.04%)

**Table A4:** Misclassification rate for men and women along with the 95% CI (12% Rounding).

		Rounded Risk		
	Original Risk	<5%	5–15%	>15%
Women	<5%	99.90% (98.88–99.91%)	0.10% (0.09–0.12%)	0.00%
	5–15%	0.53% (0.44–0.63%)	99.44% (99.33–99.53%)	0.03% (0.02–0.05%)
	>15%	0.00%	0.74% (0.49–1.06%)	99.26% (98.94–99.51%)
Men	<5%	99.82% (99.80–99.84%)	0.18% (0.16–0.20%)	0.00%
	5–15%	0.43% (0.43–0.48%)	99.50% (99.44–99.55%)	0.08% (0.06–0.10%)
	>15%	0.00%	0.71% (0.70–0.93%)	99.29% (99.07–99.46%)

## References

[1] Wright I.S., Schneider R.F., Ungerleider H.E. (1938). Factors of error in blood pressure readings. Am. Heart J..

[2] Wu C.-Y., Hu H.-Y., Chou Y.-J., Huang N., Chou Y.-C., Li C.-P. (2015). High blood pressure and all-cause and cardiovascular disease mortalities in community-dwelling older adults. Medicine.

[3] Handler J. (2009). The Importance of Accurate Blood Pressure Measurement. Perm. J..

[4] Stergiou G.S., Alpert B.S., Mieke S., Wang J., O’Brien E. (2018). Validation protocols for blood pressure measuring devices in the 21st century. J. Clin. Hypertens..

[5] Meidert A.S., Saugel B. (2018). Techniques for non-invasive monitoring of arterial blood pressure. Front. Med..

[6] Ogedegbe G., Pickering T. (2010). Principles and Techniques of Blood Pressure Measurement. Cardiol. Clin..

[7] Gulati M., Peterson L.-A., Mihailidou A. (2021). Assessment of blood pressure skills and belief in clinical readings. Am. J. Prev. Cardiol..

[8] Jones D.W., Appel L.J., Sheps S.G., Roccella E.J., Lenfant C. (2003). Measuring Blood Pressure Accurately New and Persistent Challenges. JAMA.

[9] Zhou D., Xi B., Zhao M., Wang L., Veeranki S.P. (2018). Uncontrolled hypertension increases risk of all-cause and cardiovascular disease mortality in US adults: The NHANES III Linked Mortality Study. Sci. Rep..

[10] Muntner P., Shimbo D., Carey R.M., Charleston J.B., Gaillard T., Misra S., Myers M.G., Ogedegbe G., Schwartz J.E., Townsend R.R. (2019). Measurement of blood pressure in humans: A scientific statement from the american heart association. Hypertension.

[11] (2018). Non-Invasive Sphygmomanometers—Part 2: Clinical Investigation of Intermittent Automated Measurement Type.

[12] Tang O., Juraschek S.P., Appel L.J., Cooper L.A., Charleston J., Boonyasai R.T., Carson K.A., Yeh H., Miller E.R. (2018). Comparison of automated clinical and research blood pressure measurements: Implications for clinical practice and trial design. J. Clin. Hypertens..

[13] Wen S.W., Kramer M.S., Hoey J., Hanley J.A., Usher R.H. (1993). Terminal digit preference, random error, and bias in routine clinical measurement of blood pressure. J. Clin. Epidemiology.

[14] Wingfield D., Freeman G.K., Bulpitt C.J. (2002). Selective recording in blood pressure readings may increase subsequent mortality. QJM.

[15] Broad J., Wells S., Marshall R., Jackson R. (2007). Zero end-digit preference in recorded blood pressure and its impact on classification of patients for pharmacologic management in primary care—PREDICT-CVD–6. Br. J. Gen. Pract..

[16] Ayodele O., Sanya E., Okunola O., Akintunde A. (2012). End digit preference in blood pressure measurement in a hypertension specialty clinic in southwest Nigeria. Cardiovasc. J. Afr..

[17] Anderson K.M., Odell P.M., Wilson P.W.F., Kannel W.B., Framingham M. (1991). Cardiovascular disease risk profiles. Am. Heart J..

[18] Williams B., Poulter N.R., Brown M.J., Davis M., McInnes G.T., Potter J.F., Sever P.S., Thom S.M. (2004). Guidelines for management of hypertension: Report of the fourth working party of the British Hypertension Society, 2004—BHS IV. J. Hum. Hypertens..

[19] British Cardiac Society, British Hypertension Society, Diabetes UK, HEART UK, Primary Care Cardiovascular Society (2005). Stroke Association Joint British Societies’ guidelines on prevention of cardiovascular disease in clinical practice. Heart.

[20] Brindle P.M., McConnachie A., Upton M.N., Hart C.L., Davey Smith G., Watt G.C. (2005). The accuracy of the Framingham risk-score in different socioeconomic groups: A prospective study. Br. J. Gen. Pract..

[21] Pylypchuk R., Wells S., Kerr A., Poppe K., Riddell T., Harwood M., Exeter D., Mehta S., Grey C., Wu B.P. (2018). Cardiovascular disease risk prediction equations in 400 000 primary care patients in New Zealand: A derivation and validation study. Lancet.

[22] Te Whatu Ora (2003). The Assessment and Management of Cardiovascular Risk. New Zealand Guidelines Group. https://www.tewhatuora.govt.nz/assets/Publications/Cardiovascular-Publications/assessment-and-management-of-cardiovascular-risk-full.pdf.

[23] Cholesterol Treatment Trialists’ (CTT) Collaborators (2012). The effects of lowering LDL cholesterol with statin therapy in people at low risk of vascular disease: Meta-analysis of individual data from 27 randomised trials. Lancet.

[24] The Blood Pressure Lowering Treatment Trialists’ Collaboration (2014). Blood Pressure-Lowering Treatment Based on Cardiovascular Risk: A Meta-Analysis of Individual Patient Data. www.thelancet.com.

[25] Ministry of Health (2018). Cardiovascular Disease Risk Assessment and Management for Primary Care.

[26] Helmreich J.E. (2015). Regression Modeling Strategies with Applications to Linear Models, Logistic and Ordinal Regression and Survival Analysis.

[27] PHARMAC (2020). Cost Resource Manual. https://pharmac.govt.nz/medicine-funding-and-supply/the-funding-process/policies-manuals-and-processes/economic-analysis/cost-resource-manual#s12.

[28] PHARMAC (2024). Community Schedule. https://schedule.pharmac.govt.nz/2024/10/01/Schedule.pdf.

[29] bpac (2023). Oral Anticoagulant Selection in Primary Care. https://bpac.org.nz/2023/docs/anticoagulants.pdf.

[30] bpac (2024). Hypertension in Adults: The Silent Killer. https://bpac.org.nz/2023/hypertension.aspx.

[31] bpac (2021). Prescribing Statins to Reduce Cardiovascular Risk. https://bpac.org.nz/2021/statins.aspx.

[32] bpac (2011). The Use of Antithrombotics Medicines in General Practice. https://bpac.org.nz/bpj/2011/october/docs/bpj_39_antithrombotic_pages_10-21.pdf.

[33] Canoy D., Copland E., Nazarzadeh M., Ramakrishnan R., Pinho-Gomes A.-C., Salam A., Dwyer J.P., Farzadfar F., Sundström J., Woodward M. (2022). Antihypertensive drug effects on long-term blood pressure: An individual-level data meta-analysis of randomised clinical trials. Heart.

[34] Lv H.-L., Jin D.-M., Liu M., Liu Y.-M., Wang J.-F., Geng D.-F. (2014). Long-term efficacy and safety of statin treatment beyond six years: A meta-analysis of randomized controlled trials with extended follow-up. Pharmacol. Res..

[35] BPJ (2010). An Update on Statins. https://bpac.org.nz/bpj/2010/august/statins.aspx.

[36] bpac (2018). Cardiovascular Disease Risk Assessment in Primary Care: Managing Lipids. https://bpac.org.nz/2018/docs/lipids.pdf.

[37] Hamrahian S.M., Maarouf O.H., Fülöp T. (2022). A Critical Review of Medication Adherence in Hypertension: Barriers and Facilitators Clinicians Should Consider. Patient Prefer. Adherence.

[38] Beevers G., Lip G.Y.H., O’Brien E. (2001). Blood pressure measurement: Part II—Conventional sphygmomanometry: Technique of auscultatory blood pressure measurement. BMJ.

[39] Nietert P.J., Wessell A.M., Feifer C., Ornstein S.M. (2006). Effect of terminal digit preference on blood pressure measurement and treatment in primary care. Am. J. Hypertens..

[40] (2002). Manual, Electronic, or Automated Sphygmomanometers.

[41] de Lusignan S., Belsey J., Hague N., Dzregah B. (2004). End-digit preference in blood pressure recordings of patients with ischaemic heart disease in primary care. J. Hum. Hypertens..

[42] Ostchega Y., Prineas R.J., Paulose-Ram R., Grim C.M., Willard G., Collins D. (2003). National Health and Nutrition Examination Survey 1999–2000: Effect of observer training and protocol standardization on reducing blood pressure measurement error. J. Clin. Epidemiology.

[43] Karmali K.N., Lloyd-Jones D.M. (2017). Global risk assessment to guide blood pressure management in cardiovascular disease prevention. Hypertension.

[44] Armstrong R.S. (2002). Nurses’ knowledge of error in blood pressure measurement technique. Int. J. Nurs. Pract..

[45] Badawy M.A.E.M.D., Naing L., Johar S., Ong S., Rahman H.A., Tengah D.S.N.A.P., Chong C.L., Tuah N.A.A. (2022). Evaluation of cardiovascular diseases risk calculators for CVDs prevention and management: Scoping review. BMC Public Heal..

[46] García-Fernández-Bravo I., Torres-Do-Rego A., López-Farré A., Galeano-Valle F., Demelo-Rodriguez P., Alvarez-Sala-Walther L.A. (2022). Undertreatment or Overtreatment With Statins: Where Are We?. Front. Cardiovasc. Med..

[47] Bress A.P., Colantonio L.D., Cooper R.S., Kramer H., Booth J.N., Odden M.C., Bibbins-Domingo K., Shimbo D., Whelton P.K., Levitan E.B. (2019). Potential Cardiovascular Disease Events Prevented with Adoption of the 2017 American College of Cardiology/American Heart Association Blood Pressure Guideline. Circulation.

[48] Chen C., Li X., Su Y., You Z., Wan R., Hong K. (2022). Adherence with cardiovascular medications and the outcomes in patients with coronary arterial disease: “Real-world” evidence. Clin. Cardiol..

[49] Pickering T.G., Hall J.E., Appel L.J., Falkner B.E., Graves J.W., Hill M.N., Jones D.W., Kurtz T., Sheps S.G., Roccella E.J. (2005). Recommendations for blood pressure measurement in humans: An AHA scientific statement from the Council on High Blood Pressure Research Professional and Public Education Subcommittee. J. Clin. Hypertens..

